# Weekly administration of sagopilone (ZK-EPO), a fully synthetic epothilone, in patients with refractory solid tumours: results of a phase I trial

**DOI:** 10.1038/sj.bjc.6605327

**Published:** 2009-09-22

**Authors:** D Arnold, W Voigt, P Kiewe, C Behrmann, S Lindemann, S Reif, H Wiesinger, M Giurescu, E Thiel, H-J Schmoll

**Affiliations:** 1 Department of Hematology and Oncology, Martin Luther University, Ernst-Grube-Str., Halle/Saale 06120, Germany; 2 Department of Hematology, Oncology and Transfusion Medicine, Charité Campus Benjamin Franklin, Hindenburgdamm 30/31, Berlin 12200, Germany; 3 Department of Radiology, Martin Luther University, Halle 06097, Germany; 4 Bayer Schering Pharma AG, Mullerstr. 178, Berlin 13353, Germany; 5 Global Medical Development Oncology, Bayer Schering Pharma AG, Mullerstrasse 178, Berlin 13353, Germany

**Keywords:** dose-limiting toxicity, epothilone, maximum tolerated dose, phase I, refractory solid tumours, sagopilone

## Abstract

**Background:**

Epothilones are a novel class of microtubule-stabilising agents, and sagopilone is a fully synthetic epothilone that has shown marked *in vivo* and *in vitro* preclinical activity.

**Methods:**

This phase I, open-label study investigated the maximum tolerated dose (MTD) and dose-limiting toxicities (DLTs) of weekly sagopilone. Twenty-three patients with malignancy resistant or refractory to standard treatment were enrolled into this study evaluating sagopilone doses from 0.6 to 7.0 mg m^−2^.

**Results:**

The incidence of drug-related haematological adverse events (AEs) was low, with two grade 3 events observed. Nonhaematological AEs were generally mild and reversible; increased *γ*-GT was the only grade 4 event and grade 3 events comprised peripheral neuropathy (*n*=2), diarrhoea (*n*=1) and fatigue (*n*=1). Two grade 3 events were DLTs (diarrhoea and peripheral neuropathy at 7.0 mg m^−2^). The MTD of weekly sagopilone was therefore established as 5.3 mg m^−2^. Stable disease was the best overall response (*n*=3). Microtubule bundle formation in peripheral blood mononuclear cells increased post-treatment, peaking after 1 h. Sagopilone disposition was similar across treatment courses and showed rapidly decreasing serum concentrations after infusion end and a long terminal disposition phase with no obvious accumulation in the serum, probably reflecting a fast uptake into tissues followed by a slow release.

**Conclusion:**

Weekly administration of sagopilone could represent an alternative to the 3-weekly administration currently evaluated in phase II trials.

Microtubules are a well-established target for antineoplastic drugs, and microtubule stabilisation by agents such as taxanes blocks cell division and induces apoptosis. This has led to widespread use of taxanes in cancer therapy (Goldspiel, 1997), although intrinsic or acquired tumour resistance limit their benefits in many patients (Horwitz *et al*, 1993).

The epothilones, a novel class of microtubule-stabilising agents, have been developed following favourable microtubule stabilisation *in vitro* (Chou *et al*, 1998), ultimately leading to the production of sagopilone, a fully synthetic epothilone (Klar *et al*, 2006), with high *in vivo* and *in vitro* activity and evasion of p-glycoprotein efflux pumps (Klar *et al*, 2006; Hoffmann *et al*, 2008).

In this phase I trial, we evaluated the safety, tolerability and pharmacokinetics of sagopilone in patients with heavily pretreated solid tumours, to determine the maximum tolerated dose (MTD) and dose-limiting toxicities (DLTs) of a weekly treatment schedule.

## Materials and methods

### Study design

This was a two-centre, open-label, phase I, non-randomised study to determine the MTD and DLTs of weekly sagopilone administration in patients with histologically confirmed malignancies. Secondary objectives were to evaluate the safety, tolerability, pharmacokinetics and pharmacodynamics of sagopilone. The study was approved by the appropriate Independent Ethics Committee and was conducted in accordance with the Declaration of Helsinki and the ICH-GCP Guidelines of 17 January 1997.

### Patient selection

Patients aged more than 18 years with histologically or cytologically confirmed malignancy that was resistant or refractory to standard treatment were included further to informed consent. Patients were required to have a World Health Organization (WHO) performance status score of 0–2, an estimated life expectancy of ⩾3 months, and adequate haematological, hepatic, renal and cardiac function. Patients were excluded from the study if they had received biological therapy, chemotherapy or immunotherapy within 4 weeks before enrolment, or radiation therapy or major surgery within 2 weeks before enrolment. Patients were also excluded if they had uncontrolled disease, current peripheral neuropathy (National Cancer Institute (NCI) common toxicity criteria grade 2), active metastatic brain disease or primary brain tumours.

### Treatment regimen

Sagopilone (10.5 mg per vial or 5.5 mg per vial lyophilised powder) was supplied by Bayer Schering Pharma AG, Berlin, Germany. For the 0.6 mg m^−2^ treatment group, one treatment course was represented by six consecutive weekly 30-min intravenous infusions, followed by a 3-week recovery period; in all other treatment groups, one treatment course consisted of three consecutive weekly intravenous infusions, followed by a 1-week recovery period). The study protocol was amended after the 0.6 mg m^−2^ group was treated to produce a more practical schedule, with shorter treatment periods and shorter, more frequent recovery periods. Treatment was to continue until disease progression or DLT (assessed after the first treatment course; therefore, after either six or three sagopilone administrations depended on the treatment group), with follow-up for 3 weeks after the last treatment.

For dose escalation, a modified phase I 3+3 Fibonacci design starting at 0.6 mg m^−2^ body surface area was used and doses were to be increased to 1.2, 1.8, 2.4, 4.0, 5.3, 7.0, 9.3 and 12.4 mg m^−2^ in each successive treatment group. The choice of the 0.6 mg m^−2^ starting dose was based on *in vivo* toxicity studies (1 out of the 10 of the MTD from the most sensitive toxicity study). As the first dose group was being completed, preliminary data from a parallel study (Schmid *et al*, 2005) indicated that dose levels of ⩽12 mg m^−2^ every 3 weeks were well tolerated. The study protocol was therefore amended to omit the 1.2, 1.8 and 2.4 doses and to continue at an equivalent dose of 4.0 mg m^−2^. Dose-limiting toxicity assessment was performed after each treatment course.

Once a patient had successfully completed an entire treatment course (after either six or three sagopilone administrations depended on the treatment group) and associated procedures, they were eligible for dose escalation. Three evaluable patients were required in each dose group, expanded to six evaluable patients if a DLT was observed in one of the three original patients. If a DLT was observed in two of the six patients in the expanded group, dose escalation was terminated and the sample size of the dose level below was increased to six patients. This lower dose was considered to be the MTD if one or more of these six patients experienced a DLT.

### Safety and tolerability

Baseline safety assessments included a physical and neurological examination, electrocardiogram (standard and digital 12-lead Holter, Teltow, Germany), chest X-ray, WHO performance status, vital signs, toxicity grading and collection of blood and urine samples for laboratory examination. These assessments were undertaken before every application of each treatment course and at regular intervals throughout the treatment course. Data were evaluated at the end of every treatment course. Toxicity and adverse events (AEs) were recorded throughout treatment and during follow-up and graded using the NCI/National Institute of Health Common Toxicity Criteria version 2.0.

### Pharmacodynamics and efficacy

Tumour evaluation was performed using WHO/International Union Against Cancer (UICC) criteria for patients with measurable disease between days 43 and 50 of each treatment course for the 0.6 mg m^−2^ treatment group, or between day 22 of the previous course and the first day of the subsequent treatment course for other dose levels. Tumour markers could be considered as evidence of measurable disease in prostate cancer (prostate-specific antigen) and in ovarian cancer (CA-125). Microtubule bundle formation in peripheral blood mononuclear cells (PBMCs) was visualised by immunofluorescent staining using 8 ml blood samples taken before, and 1 and 24 h after the end of drug administration at the first and third application of the first treatment course. Immunofluorescent staining was performed by HistoGeneX (Edegem, Belgium) using a method similar to that previously described (McDaid *et al*, 2002).

### Pharmacokinetics

Blood samples for pharmacokinetic analysis were taken during all applications in the first treatment course at baseline, then 5 and 15 min after infusion start and just before infusion end at 30 min. Further samples were collected at 5, 15, 30 and 60 min, and 2, 3, 5, 10, 24, 48, 72 and 96 h after the end of each infusion and 168 h after the end of the third infusion. For subsequent treatment courses, sampling was performed at baseline, 72 h after the end of infusion and 168 h after the end of the third infusion. Sagopilone serum concentrations were determined by validated liquid chromatography/mass spectrometry with a 0.1 ng ml^−1^ lower limit of quantitation. Pharmacokinetic evaluation was based on individual sagopilone serum concentration/time values, and parameters were calculated using the Kinetica software tool (Thermo Fisher Scientific, Philadelphia, PA, USA) without recourse to model assumptions. Individual treatment courses were only included in evaluations if ⩾1 valid point was available to assess maximum serum concentration (*C*_max_). If ⩾1 measurement was available during infusion, but not after infusion, *C*_max_ and area under the curve (AUC) measurements were estimated but not included in the statistical analysis. For the calculation of pharmacokinetic parameters other than *C*_max_ and *t*_max_, ⩾3 consecutive data points were required.

### Statistical analysis

Descriptive statistics were used and the number of patients, mean, s.d. and range were calculated for the relevant variables. For pharmacodynamics assessments, repeated-measurement models evaluating the relationship of microtubule bundling (percentage of affected PBMCs) with time point or dosage (mg m^−2^) were calculated with PROC GLM in SAS (v 9.1; SAS Institute Inc., Cary, NC, USA).

## Results

### Patient characteristics

Of 24 patients screened at two German centres, 23 were enrolled in the study between January 2004 and November 2005. All 23 patients received treatment and were evaluable for safety, 20 patients comprised the per-protocol set and 11 patients had samples evaluable for pharmacokinetics. Patients with a variety of primary tumour types, including pleural mesothelioma, colorectal cancer, renal cell cancer, breast cancer and choroidal melanoma, were enrolled in the study (Table 1). Most patients (87%) had a WHO status ⩽1 at baseline. Patients had undergone a high level of pretreatment; 96% of patients had received previous chemotherapy, including previous taxane treatment in 43% and previous cytokine treatment in 4%. The median (range) number of previous chemotherapy regimens was 4 (1–14). Eight patients had previously received radiotherapy, whereas 16 patients had undergone earlier surgery. Despite this level of pretreatment, patients had no, or a low, response to earlier therapy (the majority classified as progressive disease).

### Treatment and dosing

Of the 23 patients who received study treatment, nine were assigned to the 0.6 mg m^−2^ dose, three to the 4 mg m^−2^ dose, seven to the 5.3 mg m^−2^ dose and four to the 7.0 mg m^−2^ dose. Two patients completed the study as planned, one patient receiving four and the other six treatment courses (12 and 18 sagopilone administrations, respectively), whereas 21 patients discontinued study treatment prematurely (receiving a median of 6 (range 2–18) sagopilone infusions). Of these, 14 withdrew because of disease progression, six because of AEs and one withdrew consent. Nine patients were recruited to the 0.6 mg m^−2^ dose group to have three patients that were evaluable for dose-escalation analysis. Of these nine patients, three accidentally received a reduced dose of sagopilone because of a drug preparation error in the pharmacy and were therefore excluded from the per-protocol population. Seven courses (6-weekly sagopilone treatments followed by a 3-week recovery phase) were received in the 0.6 mg m^−2^ dose group, with a median (range) of 1 (0–2), and 28 courses (3-weekly sagopilone treatments followed by 1-week recovery) were administered in the 4.0–7.0 mg m^−2^ dose groups with a median (range) of 2 (0–6).

No DLTs were reported at doses 0.6–5.3 mg m^−2^. Two DLTs were observed at 7.0 mg m^−2^: grade 3 diarrhoea and grade 3 peripheral neuropathy, experienced by one patient each (Table 2). Additional patients were therefore assigned to the lower-dose group (5.3 mg m^−2^) to a total of six evaluable for DLT analysis (one patient was not evaluable). The MTD was established as 5.3 mg m^−2^ and the highest dose given to patients was 7.0 mg m^−2^.

### Tolerability

The incidence of drug-related haematological AEs was low, with no grade 4 events and one patient each experiencing grade 3 lymphopenia and thrombocytopenia (0.6 mg m^−2^ dose) (Tables 2 and 3). Correlation of haematological AEs with serum saglopilone concentration was not possible due to the limited availability of serum samples. Drug-related nonhaematological AEs were generally mild and reversible (Tables 2 and 3) with grade 3 events, including DLTs detailed above, reported in only four patients (peripheral neuropathy in one patient each in the 5.3 and 7.0 mg m^−2^ groups, and diarrhoea and fatigue in one patient each at 7.0 mg m^−2^ sagopilone) (Table 2). One patient had a grade 4 event (increase in *γ*-GT at 7.0 mg m^−2^ sagopilone). Peripheral neuropathy was the most clinically relevant nonhaematological AE, reported in 10 patients (43.5%; one patient experienced both polyneuropathy and hypoesthesia) and considered a DLT in one patient (Tables 2 and 3). The estimated median time to neuropathy onset was 18 days. At baseline, nine patients had grade 1 peripheral neuropathy, five of whom had previous taxane treatment. During this study, the majority of the peripheral neuropathy events observed were grades 1 and 2 (eight patients), and no grade 4 events were reported. Grades 1 and 2 treatment-related nausea and vomiting were observed in 22 and 17% of patients, respectively (Table 3). The only occurrence of diarrhoea was as a DLT (grade 3) at 7.0 mg m^−2^ sagopilone (Table 2). No hypersensitivity reactions were reported, despite the lack of premedication, and no patients died while receiving sagopilone treatment.

### Pharmacodynamics and efficacy

Post-treatment tumour evaluation data are available for 11 patients; 12 patients were excluded due to incorrect dosing and absence of post-treatment data (2 patients and 10 patients, respectively). No objective responses were observed in this heavily pretreated population and stable disease was the best overall response, reported in three patients: one with cholangiocellular carcinoma (4.0 mg m^−2^ dose), and one each with fallopian tube cancer and pleural mesothelioma (5.3 mg m^−2^ dose). Follow-up data were available for 10 patients in groups 4.0–7.0 mg m^−2^, and of the patients who achieved stable disease, the median (range) of stable disease duration was 68.5 (23–162) days.

**Figure 1:**
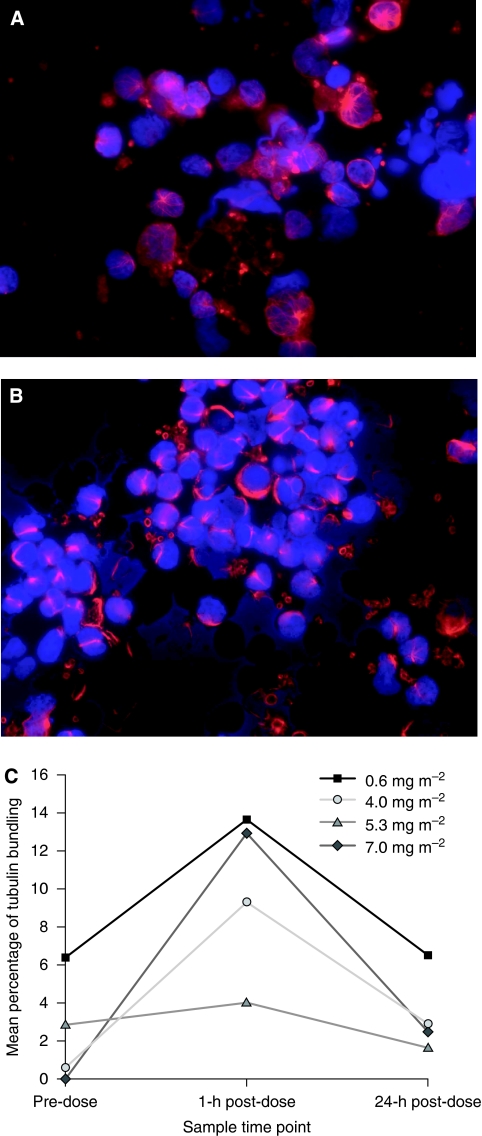
Visualisation of microtubule bundle formation in peripheral blood mononuclear cells in a patient sample from the 7.0 mg m^−2^ dose group (**A**) before treatment, (**B**) 1 h after drug application and (**C**) microtubule bundle formation over time with sagopilone treatment before 1 h and 24 h after the first application of sagopilone during the first treatment course.

Complete data sets (pre-dose, 1 h post-dose and 24 h post-dose samples evaluable) of PBMC tubulin immunofluorescent staining were available for 19 patients. Peripheral bundle formation was increased in post-treatment PBMC samples taken during the first treatment course (Figure 1A, B) and was highest 1 h after treatment application (Figure 1C), reaching a maximum overall mean±s.d. increase from pre-dose levels of 7.61±10.15% for the first sagopilone application. This was also observed to a lesser extent for the third sagopilone application of the first course, with a maximum overall mean ±s.d. increase from pre-dose levels of 5.84±7.40%. A repeated-measurement model indicated that the time of sample collection, but not the dose level, was related to the degree of tubulin bundle formation (*P*=0.0058 and 0.2299, respectively).

### Pharmacokinetics

**Figure 2:**
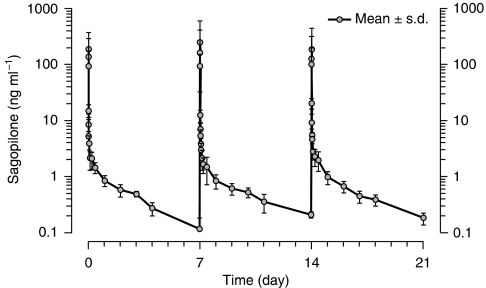
The mean concentration–time profile of sagopilone administered weekly at 5.3 mg m^−2^ over three treatment cycles.

Pharmacokinetic data are available for 11 patients. Mean pharmacokinetic parameters are presented for the 4.0 and 5.3 mg m^−2^ treatment groups only, as data from only one and two patients were available for the 0.6 and 7.0 mg m^−2^ groups, respectively (Table 4). The largest number of evaluable patients (*n*=6) were in the 5.3 mg m^−2^ group. For treatment cycles 1 and 3, the mean AUC (0–*t*_last_) was between 153 and 157 ng h^−1^ ml^−1^, the mean terminal elimination half-life (*t*_1/2_) ranged from 33 to 58 h, the mean total body clearance was between 1000 and 1359 ml min^−1^, and the mean volume of distribution at steady state (*V*_ss_) ranged from 1851 to 4852 l (Table 4). The large distribution volume indicates high tissue and/or tubulin binding. The mean concentration–time profile of sagopilone showed a multiexponential disposition (Figure 2). Sagopilone serum concentrations decreased to <10% peak concentration by 10 h after infusion end, followed by a long terminal disposition phase. The pharmacokinetics of sagopilone at the 5.3 mg m^−2^ dose were similar during these three applications. No obvious differences in the pharmacokinetics (e.g., *C*_max_ or AUC) could be seen between treatment courses, suggesting that no or only minimal accumulation occurred. Owing to small sample sizes and methodological difficulties related to the collection of plasma samples, any pharmacokinetic data should be interpreted with caution and no correlation to dose linearity or relationships between sagopilone serum concentrations and microtubule binding in PBMCs could be drawn.

## Discussion

In this phase I dose-escalation study, weekly sagopilone administration was well tolerated in heavily pretreated patients with solid tumours. Two DLTs, one case each of grade 3 peripheral neuropathy and grade 3 diarrhoea, were observed at 7.0 mg m^−2^ and the MTD was determined as 5.3 mg m^−2^. The incidence of diarrhoea associated with sagopilone use is generally low, with a similar phase I trial evaluating a once-every-3-week regimen of sagopilone administration recently reporting an MTD of 22 mg m^−2^, and a low incidence of diarrhoea (Schmid *et al*, 2005).

Peripheral neuropathy was the most clinically relevant AE and a DLT in one patient. This was not an unexpected finding in a heavily pretreated study population as neuropathy can be a cumulative toxicity from many chemotherapy compounds, and is a common AE in patients treated with microtubule-stabilising agents (Marupudi *et al*, 2007). Despite pretreatment with taxanes in 10 patients and grade 1 peripheral neuropathy in nine patients at baseline, the overall incidence of peripheral neuropathy in this investigation was moderate, with only two grade 3 events and no grade 4 events. There was no correlation between cumulative peripheral neuropathy and earlier taxane use; this ensures the MTD results from sagopilone administration alone and not a combination of sagopilone and earlier taxane use. However, as some patients may have not received several courses of earlier taxane therapy, there still remains some uncertainty in precise assessment of cumulative toxicity such as neuropathy.

The second DLT observed, grade 3 diarrhoea, affected only one patient. Diarrhoea has rarely been reported with sagopilone treatment, although it is a DLT of patupilone and is frequently observed with ixabepilone (Low *et al*, 2005; Rubin *et al*, 2005). Nausea and vomiting were mild-to-moderate and easily manageable with standard antiemetics. The incidence of haematological events was low, consistent with the other phase I sagopilone trial (Schmid *et al*, 2005) but in contrast to that seen with ixabepilone therapy, which has neutropenia as a DLT (Zhuang *et al*, 2005).

No objective responses were observed in this heavily pretreated population with tumours resistant to many anticancer treatments. This observation is unsurprising considering the small sample size and medical history of the population. Stable disease was the best response observed in three patients, one of whom had fallopian tube cancer refractory to four previous chemotherapy regimens, notably including paclitaxel.

Microtubule bundle formation in PBMCs is a marker of the ability of a microtubule-stabilising drug to bind to its target *in vivo* and induce tubulin polymerisation (Horwitz *et al*, 1993). Tubulin polymerisation was highest in samples taken 1 h after sagopilone infusion, indicating that sagopilone rapidly induces *in vivo* microtubule binding with a similar time course to that reported for ixabepilone (McDaid *et al*, 2002; Mani *et al*, 2007). The reduction of bundle formation seen 24 h post-dose may indicate that this binding process is reversible and/or may be due to the rapid clearance of sagopilone from the circulatory system.

In patients receiving 5.3 mg m^−2^ sagopilone, the pharmacokinetics of sagopilone were similar across each treatment cycle and course. The rapid initial decrease in serum concentrations after the end of infusion was possibly due to fast uptake of sagopilone into tissues. The long *t*_1/2_ most likely reflects the release of sagopilone from deep tissue compartments, rather than the rate of metabolism or excretion. This rapid uptake and slow release from tissues supports data from preclinical sagopilone research (Hoffmann *et al*, 2008).

Owing to the limited sample size, these pharmacokinetic conclusions should be interpreted with caution as, unfortunately, in some patients the intended blood sampling did not occur, for example, blood samples may have been taken a few minutes after the end of sagopilone infusion, as opposed to shortly before the end of infusion. Therefore, the rapid elimination of sagopilone (as shown in Figure 2) impeded accurate measurements of *C*_max_, and the small samples size available was not able to overcome these operational challenges.

This study shows that 5.3 mg m^−2^ sagopilone administered weekly is feasible and well tolerated. However, the 3-weekly administration reported by Schmid *et al* (2005), with an MTD of 22 mg m^−2^, was considered to be the preferable regimen due to its tolerability profile and more convenient dosing schedule. Encouraging results from both phase I trials have led to a broad phase II programme, with ongoing trials using the 3-weekly schedule in several indications, including prostate, ovarian, breast and lung cancer, glioblastoma and melanoma. Proof-of-concept has been established for sagopilone in platinum-resistant (Rustin *et al*, 2007a) and platinum-sensitive (McMeekin *et al*, 2008) ovarian cancer, melanoma (Wenk *et al*, 2008) and androgen-independent prostate cancer (Graff *et al*, 2008). Results from additional trials will provide further evidence of the clinical potential of sagopilone.

## Figures and Tables

**Table 1:** Patient demographics, disease status and earlier neoplastic treatment

**Characteristic**	**0.6 mg m^−2^ (*n*=9)**	**4.0 mg m^−2^ (*n*=3)**	**5.3 mg m^−2^ (*n*=7)**	**7.0 mg m^−2^ (*n*=4)**
*Age, n*				
Mean	52	41	55	41
Range	44–65	33–68	48–67	25–70
				
*Sex, n*				
Female	3	3	3	2
Male	6	0	4	2
				
*WHO performance status, n*				
0	5	1	2	2
1	4	1	4	2
2	0	1	1	0
*Tumour types*	Pancreatic cancer (two patients), retroperitoneal cancer, breast cancer, colon cancer, thymus cancer, choroid melanoma, adrenal gland cancer, peritoneal cancer	Urothelial cancer, cholangiocellular carcinoma, renal sarcoma	Pancreatic cancer, pleural mesothelioma (two patients), gallbladder carcinoma, fallopian tube cancer, renal cell carcinoma, rectal cancer	Gallbladder carcinoma, cancer of unknown primary origin, oesophageal cancer, epithelioid sarcoma
				
*Previous therapy, n*				
Chemotherapy	9	3	6	4
Taxanes	4	1	3	2
Immunotherapy	2	1	3	1
Hormone therapy	1	0	1	0
Radiotherapy	5	1	1	1
Surgery	6	3	5	2
				
*Number of earlier chemotherapy regimens, n*				
0	0	0	1	0
1	1	0	1	0
2	2	1	3	1
3	1	1	0	0
4	2	0	0	0
⩾5	3	1	2	3

**Table 2:** Grade ⩾3 drug-related adverse events and dose-limiting toxicities

**Adverse event**	**0.6 mg m^−2^**, ***n* (*n*=9)**	**4.0 mg m^−2^, *n* (*n*=3)**	**5.3 mg m^−2^, *n* (*n*=7)**	**7.0 mg m^−2^, *n* (*n*=4)**	**Overall, *n* (*n*=23)**
Peripheral neuropathy			1	1^a^	2 (9)
Diarrhoea				1^a^	1 (4)
Fatigue				1	1 (4)
Increased *γ*-GT				1^b^	1 (4)
Lymphopenia	1				1 (4)
Thrombocytopenia	1				1 (4)

a Dose-limiting toxicities; all events grade 3 except grade 4.

b Grade 4.

**Table 3:** Grades 1 and 2 drug-related adverse events (AEs)

**AE**	**0.6 mg m^−2^, *n* (*n*=9)**	**4.0 mg m^−2^, *n* (*n*=3)**	**5.3 mg m^−2^, *n* (*n*=7)**	**7.0 mg m^−2^, *n* (*n*=4)**	**Overall, *n* (%) (*n*=23)**
*Haematological AEs*					
Lymphopenia	1	1	1		3 (13)
Decreased haemoglobin	1	1	2	1	5 (22)
					
*Nonhaematological AEs*					
Nausea	2	1	1	1	5 (22)
Vomiting	2	1		1	4 (17)
Constipation			1	1	2 (9)
Abdominal pain				2	2 (9)
Peripheral neuropathy	4	1	2	1	8 (35)
Hyperesthesia				1	1 (4)
Asthenia				1	1 (4)
Eye irritation			1		1 (4)
Headache		1			1 (4)
Intention tremor			1		1 (4)
Mucosal inflammation	1				1 (4)
Peripheral oedema			1		1 (4)
Pyrexia				1	1 (4)
Subileus				1	1 (4)
Temperature intolerance			1		1 (4)

**Table 4:** Summary of mean pharmacokinetic parameters and mean dose-independent pharmacokinetic parameters of sagopilone at 4.0 and 5.3 mg m^−2^ doses

**Dose group (mg m^−2^)**	**Total dose (mg)**	**Treatment cycle**	***C*_max_ (ng ml^−1^)**	***t*_max_ (h)^a^**	**AUC (0–*t*_last_) (ng h^−1^ ml^−1^)**	**AUC (ng h^−1^ ml^−1^)**	***t*_1/2_ v (h)**	**CL (ml min^−1^)**	***V*_ss_ (l)**
4 (*n*=3)	6.85 (17.0%)	1 (day 1)	67.5 (218%) (2)	0.5–0.58 (2)	NA	400 (1)	46.3 (1)	247 (1)	313 (1)
		2 (day 8)	79.0 (650%)	0.5 (0.08–0.55)	92.5 (1)	NA	NA	NA	NA
		3 (day 15)	13.5 (1)	0.5 (1)	NA	NA	NA	NA	NA
5.3 (*n*=6)	9.89 (8.59%)	1 (day 1)	188 (97.5%) (4)	0.25 (0.17–0.5)	153 (41.4%) (4)	133 (33%) (2)	57.8 (14.1%) (3)	1359 (26.7%) (3)	4852 (50%) (3)
		2 (day 8)	128 (144%)	0.5 (0.08–0.5)	166 (48.8%) (5)	161 (1)	65.3 (1)	878 (1)	4964 (1)
		3 (day 15)	101 (213%) (5)	0.5 (0.25–0.52)	157 (42.1%) (4)	138 (7.26%) (2)	33.3 (49.4%) (3)	1000 (19.3%) (3)	1851 (65%) (3)

All values shown are geometric mean (geometric coefficients of variation) except median (range).

a Median (range); (*n*)=number of patients with evaluable PK parameters; *C*_max_=maximal serum concentration following treatment on days 1, 8 and 15; *t*_max_=time to reach maximal serum concentration following treatment on days 1, 8 and 15; AUC(0–*t*_last_)=area under the serum concentration–time curve from 0-h data point up to last data point > lower limit of quantitation; AUC=total area under the serum concentration-time curve from 0-h data point up to infinity; *t*_1/2_=terminal elimination half-life; CL=total body clearance; *V*_ss_=volume of distribution at steady state; NA=not applicable.

## References

[bib1] Chou T-C, Zhang X-G, Balog A, Su D-S, Meng D, Savin K, Bertino JR, Danishefsky SJ (1998) Desoxyepothilone B: an efficacious microtubule-targeted antitumor agent with a promising *in vivo* profile relative to epothilone B. Proc Natl Acad Sci USA 95: 9642–9647 9689134 10.1073/pnas.95.16.9642 PMC21392

[bib2] Goldspiel BR (1997) Clinical overview of the taxanes. Pharmacotherapy 17: 110S–125S 9322878

[bib3] Graff J, Smith DC, Neerukonda L, Alonso M, Edgar A, Wang Y, Beer TM, The Prostate Cancer Clinical Trials Consortium (2008) Phase II study of sagopilone (ZK-EPO) plus prednisone as first-line chemotherapy in patients with metastatic androgen-independent prostate cancer 44th ASCO Annual Meeting, Chicago, Illinois, USA, May 30–June 3, Poster

[bib4] Hoffmann J, Vitale I, Buchmann B, Galluzzi L, Schwede W, Senovilla L, Skuballa W, Vivet S, Lichtner RB, Vicencio JM, Panaretakis T, Siemeister G, Lage H, Nanty L, Hammer S, Mittelstaedt K, Winsel S, Eschenbrenner J, Castedo M, Demarche C, Klar U, Kroemer G (2008) Improved cellular pharmacokinetics and pharmacodynamics underlie the wide anticancer activity of sagopilone. Cancer Res 68: 5301–5308 18593931 10.1158/0008-5472.CAN-08-0237

[bib5] Horwitz SB, Cohen D, Rao S, Ringel I, Shen H-J, Yang C-PH (1993) Taxol: mechanisms of action and resistance. J Natl Cancer Inst Monogr 15: 55–61 7912530

[bib6] Klar U, Buchmann B, Schwede W, Skuballa W, Hoffmann J, Lichtner RB (2006) Total synthesis and anti-tumor activity of ZK-EPO: the first fully synthetic epothilone in clinical development. Angew Chem Int Ed Engl 45: 7942–7948 17006870 10.1002/anie.200602785

[bib7] Low JA, Wedam SB, Lee JJ, Berman AW, Brufsky A, Yang SX, Poruchynsky MS, Steinberg SM, Mannan N, Fojo T, Swain SM (2005) Phase II clinical trial of ixabepilone (BMS-247550), an epothilone B analog, in metastatic and locally advanced breast cancer. J Clin Oncol 23: 2726–2734 15837987 10.1200/JCO.2005.10.024

[bib8] Mani S, McDaid HM, Grossman A, Muggia F, Goel S, Griffin T, Colevas D, Horwitz SB, Egorin MJ (2007) Peripheral blood mononuclear and tumor cell pharmacodynamics of the novel epothilone B analogue, ixabepilone. Ann Oncol 18: 190–195 17018704 10.1093/annonc/mdl315

[bib9] Marupudi NI, Han JE, Li KW, Renard VM, Tyler BM, Brem H (2007) Paclitaxel: a review of adverse toxicities and novel delivery strategies. Expert Opin Drug Saf 6: 609–621 17877447 10.1517/14740338.6.5.609

[bib10] McDaid HM, Mani S, Shen H-J, Muggia F, Sonnichsen D, Horwitz SB (2002) Validation of the pharmacodynamics of BMS-247550, an analogue of epothilone B, during a phase I clinical study. Clin Cancer Res 8: 2035–2043 12114401

[bib11] McMeekin S, Patel R, Verschraegen C, Celano P, Burke J, Plaxe S, Ghatage P, Giurescu M, Stredder C, Wang Y (2008) Phase I/II study of sagopilone (ZK-EPO) plus carboplatin in women with recurrent platinum-sensitive ovarian cancer. Ann Oncol 19(Suppl 8). Abstract 665O, pviii211 10.1038/bjc.2011.499 PMC3251849 22108514

[bib12] Rubin EH, Rothermel J, Tesfaye F, Chen T, Hubert M, Ho Y-Y, Hsu C-H, Oza AM (2005) Phase I dose-finding study of weekly single-agent patupilone in patients with advanced solid tumors. J Clin Oncol 23: 9120–9129 16301595 10.1200/JCO.2005.03.0981

[bib13] Rustin G, Reed N, Jayson G, Ledermann J, Adams M, Stredder C, Wagner A, Giurescu M, The ZK-EPO Study Group in Platinum-Resistant Ovarian Cancer (2007a) Phase II trial of sagopilone (ZK-EPO), a novel epothilone, in patients with platinum-resistant ovarian cancer. 15th International Meeting of the European Society for Gynaecological Oncology: Berlin, Germany, October 28–November 1, Poster

[bib14] Schmid P, Kiewe P, Kuehnhardt D, Korfel A, Lindemann S, Giurescu M, Reif S, Thiel E, Possinger K (2005) A phase I study of the novel, third generation epothilone ZK-EPO in patients with advanced solid tumors. J Clin Oncol (Meeting Abstracts) 23. Abstract 2051, p147s

[bib15] Wenk D, DeConti R, Urbas P, Andrews S, Sondak V, Maker N, Weber J, Daud A (2008). Phase II trial of sagopilone (ZK-EPO), a novel epothilone, in patients with metastatic melanoma 44th ASCO Annual Meeting, Chicago, Illinois, USA, May 30–June 3, Poster 9046

[bib16] Zhuang SH, Agrawal M, Edgerly M, Bakke S, Kotz H, Thambi P, Rutt A, Balis FM, Bates S, Fojo T (2005) A Phase I clinical trial of ixabepilone (BMS-247550), an epothilone B analog, administered intravenously on a daily schedule for 3 days. Cancer 103: 1932–1938 15800893 10.1002/cncr.20977

